# Cimifugin ameliorates ulcerative colitis-related lung injury by modulating the JAK1/STAT1 signaling pathway and macrophage M1 polarization

**DOI:** 10.3389/fimmu.2025.1551892

**Published:** 2025-07-01

**Authors:** Guanyuan Wang, Fan Yang, Guodong Zang, Ning Shen, Lina Huang, Zhaotian Ma, Ming Li

**Affiliations:** ^1^ Traditional Chinese Medicine (TCM) Rehabilitation Department, The Fifth People’s Hospital of Jinan, Jinan, China; ^2^ Department of Respiratory and Critical Care Medicine, Affiliated Hospital of Shandong University of Traditional Chinese Medicine, Jinan, China; ^3^ First College of Clinical Medicine, Shandong University of Traditional Chinese Medicine, Jinan, China; ^4^ College of Pharmacy, Jining Medical University, Jining, China; ^5^ College of Integrated Traditional Chinese and Western Medicine, Jining Medical University, Jining, China

**Keywords:** cimifugin, ulcerative colitis-related lung injurys, JAK1/STAT1, gut microbiota, macrophage

## Abstract

**Introduction:**

Ulcerative colitis (UC)-related lung injurys is a commonly overlooked extraintestinal manifestation and there are currently no drugs with definitive efficacy available. Cimifugin has been found to inhibit aberrant inflammation and oxidative stress, but its efficacy in UC-related lung injurys has not yet been demonstrated.

**Methods:**

This study explored the effects of Cimifugin on UC-related lung injurys using RNA-seq in combination with 16S rRNA sequencing.

**Results:**

Cimifugin significantly ameliorated symptoms and attenuated colon and lung injury in a UC mouse model, restored the integrity of the intestinal and lung epithelial barriers, and suppressed lung inflammation, which was achieved by inhibiting the JAK1/STAT1 pathway and the M1 macrophage-mediated inflammatory state in the colon and lungs, as well as by improving the homeostasis of the intestinal microbiota.

**Discussion:**

Cimifugin ameliorates UC-associated lung injury by modulating the JAK1/STAT1 pathway and macrophage M1 polarization.

## 1 Introduction

Inflammatory bowel diseases (IBDs) are a chronic digestive disease that afflicts millions of people worldwide and consists of Crohn disease and ulcerative colitis (UC) (1, 2). UC is more common than Crohn disease, and in addition to causing damage to the colon and rectum, 40-50% of patients often have skeletal muscle, skin, lung, and eye complications, which are called “extraintestinal manifestations” (3–6). Lung injury is an parenteral manifestation that was often overlooked in the past, but existing evidence suggests that lung injury occurs much more frequently than previously recognized, mainly because most patients do not notice their respiratory symptoms (7, 8). Lung injury in patients with UC can occur in the airways, lung parenchyma, and interstitium, but airway injury characterized by bronchodilatation and airway inflammation is the most prevalent type. The pathogenesis of UC-related lung injury is currently unknown, and the possible causes include systemic chronic inflammation triggered by immunodeficiency, dysregulation of protease activity, and impairment of intestinal barrier function (9–11). However, all these hypotheses need to be further confirmed.

Clinical treatments for UC include aminosalicylates, immunosuppressants, and biologics. However, the long-term efficacy of these drugs is mediocre and associated with varying degrees of side effects. Importantly, there is no evidence that they attenuate UC-related lung injury.These factors lead to a reduced quality of life for patients, prompting many UC patients to seek complementary and/or alternative therapies (12).Cimifugin is a coumarin derivative isolated from *Saposhnikovia divaricata*, a plant traditionally used in the treatment of digestive disorders, skin diseases, and immune disorders. Recent studies have confirmed that Cimifugin can inhibit inflammation and oxidative stress (13–15). In an irritable bowel syndrome disease model, cimifugin regulates the NRF2/HO-1 signaling pathway by up-regulating the expression of SIRT1, which effectively maintains intestinal barrier integrity and intestinal function (16). Meanwhile, cimifugin also reduced the separated gap between airway epithelial cells by regulating the expression of tight junction proteins (17).These results suggest the potential value of cimifugin application in UC-related lung injury, but have not been explored yet.

In this study, we constructed a classical UC mouse model using dextran sulfate sodium (DSS) and investigated for the first time the therapeutic effect of cimifugin on UC-related lung injury (18, 19). We further analyzed the potential mechanism of action of Cimifugin using RNA-seq and 16S rRNA sequencing, and found that cimifugin mainly affected the JAK1/STAT1 signaling pathway. In addition, we explored the possibility that cimifugin prevents macrophage M1 polarization to maintain lung and colon immune homeostasis. The findings provide a reliable data resource for further understanding of the pathogenesis of UC-related lung injury, as well as a scientific basis for the clinical application of cimifugin.

## 2 Materials and methods

### 2.1 Reagents

Cimifugin (LOT: S02HB193600, HPLC ≥98%) was purchased from Shanghai Yuanye Biotechnology Co. Mesalazine was purchased from Heilongjiang Tianhong Pharmaceutical Co. DSS(MW 36,000–50,000) was sourced from MP Biochemicals. The above reagents were stored according to the manufacturer’s recommendations and used at the specified concentrations.

### 2.2 Animal experiments and sample collection

Male C57BL/6 J mice aged 6-8 weeks were purchased from Pengyue Experimental Animal Breeding Co., LTD. (Jinan, China). The mice were kept in conditions free of specific pathogens with a humidity of 40-80%, a temperature of 24 ± 1°C, and a 12-hour light/dark cycle. The study was conducted in accordance with the Declaration of Helsinki, all animal testing procedures were approved by the Experimental Animal Ethics Committee of Shandong Academy of Traditional Chinese Medicine (No. SDZYY20230621005). After 7 days of adaptive feeding, the mice were randomly divided into 6 groups. Group 1 (normal control group): no DSS exposure and no special treatment; Group 2 (model group): DSS exposure and the same volume of normal saline intragastric treatment; Group 3 (positive control group): DSS exposure and reference treatment with 500 mg/kg mesalazine; Groups 4-6 were high (CIM-H), medium (CIM-M), and low (CIM-L) dose groups: DSS exposure and treatment with 50mg/kg, 25mg/kg, and 12.5 mg/kg of cimifugin, respectively (20). Induction of the UC model was achieved by administering 3% DSS to mice for 7 consecutive days. In short, the DSS was dissolved to a 3% concentration in drinking water and the mice were allowed to drink freely. The DSS solution is changed once a day and the corresponding medication is administered at the same time as the DSS exposure. The weight and fecal characteristics of the mice were monitored during the experiment. The Disease Activity Index (DAI) scoring in mice was performed using the following equation, according to the method of Bang et al (21). DAI = (weight loss score + stool trait score + blood in stool score)/3. After the experiment, the mice were euthanized, the colon length was measured and photographed, and the colon contents were collected for 16 S rRNA gene sequencing. Liver, kidney, part of colon and lung tissue were immobilized in 4% paraformaldehyde for 24 hours for pathology testing, while the rest of colon and lung were rapidly frozen in liquid nitrogen and stored at -80 °C for subsequent analysis.

### 2.3 Histomorphological analysis

The tissue was fixed with paraformaldehyde, then paraffin embedded, cut into 4 μm thick slices, and stained with hematoxylin and eosin (H&E). According to the methods provided in the literature, colon tissue and lung tissue were respectively scored for inflammation (22, 23).

### 2.4 Immunofluorescence

Anti-F4/80 rabbit pAb and anti-iNOS rabbit pAb were purchased from Solarbio Science & Technology Co., Ltd. (Wuhan, China). Remove wax from lung and colon tissue slices and perform antigen repair. After cooling the slices to room temperature, apply 3% bovine serum albumin for 30 minutes and incubate the primary antibody overnight at 4 °C. The primary antibodies used include F4/80 and iNOS (diluted 1:100). After washing with phosphate buffered saline, the slices were incubated with goat anti rabbit secondary antibody (diluted 1:200) at 37 °C for 50 minutes. The nucleus was re-stained with 4’, 6-diamidino-2-phenylindole, and an autofluorescence quenchant was added. The film was washed and sealed, and images were observed under a fluorescence microscope. We used Image J software to quantitatively analyze the immunofluorescence images.

### 2.5 Western blot

Rabbit anti-JAK1 antibody (GB115719), rabbit anti-P-JAK1 antibody (GB115604), rabbit anti-P-STAT1 antibody (GB115605), rabbit anti-iNOS antibody (GB113965), rabbit anti-IL-1β antibody (GB115752), rabbit anti-Arg1 antibody (GB115724), rabbit anti-Occludin antibody (GB111401), rabbit anti-ACTIN antibody (GB15003), rabbit anti-ZO-1 antibody (GB111402) and goat anti-rabbit IgG antibodies (GB23303) were purchased from Servicebio Technology Co., Ltd (Wuhan, China). Rabbit anti-STAT1 antibody (10144-2-AP) was obtained from Sanying Biotechnology Co., Ltd (Wuhan, China). Rabbit anti-CD206 antibody (DF4149) was obtained from Affinity Biosciences Co., Ltd (Jiangsu, China). Following total protein extraction, protein concentration was determined using the BCA method. Equal amounts of protein samples were subjected to gel electrophoresis and membrane transfer. After blocking, the membranes were incubated overnight at 4°C with primary antibodies against JAK1, P-JAK1, STAT1, P-STAT1, iNOS, IL-1β, Arg1, CD206, Occludin, and ZO-1 (diluted 1:1,000) in Tris-buffered saline containing Tween 20 (TBST) supplemented with 5% non-fat milk. Subsequently, a secondary goat anti-rabbit antibody (diluted 1:200) was applied and incubated at room temperature for 1.5 hours. The membranes were then washed four times with TBST to remove excess antibodies, and protein bands were visualized on the membrane. The gray values of the protein bands were detected using AlphaEase FC software.

### 2.6 16S rRNA gene sequencing and analysis

After isolation of genomic DNA of bacteria from fecal samples, PCR amplification of the V3-V4 region was performed with sequencing analysis and species identification. Operational taxonomic units (OTUs) obtained from the standard analysis were screened for samples and species, and the Alpha diversity index of the samples was calculated using Qiime software (Version1.9.1). The OTUs in the samples were sorted by relative abundance (or number of sequences contained) from largest to smallest to obtain the corresponding sort number, and then the Rank Abundance curve was plotted with the sort number of the OTUs as the horizontal coordinate and the relative abundance in the OTUs as the vertical coordinate. Beta diversity analysis was performed with Qiime software (Version 1.9.1) and non-metric multidimensional scaling (NMDS) was performed using the vegan package of R. LDA Effect Size (LEfSe) analysis was used for biomarker discovery and revealing macrogenomic features. Spearman correlation analysis was performed on all samples to obtain the species correlation coefficient matrix and then remove the connections with correlation coefficients < 0.6 to build a genus-level species correlation network diagram. Finally, Phylogenetic Investigation of Communities by Reconstruction of Unobserved States (PICRUSt) analysis was performed based on the gene information on Greengene database and OTUs, i.e., to construct the predicted gene function of microorganisms in the samples. In other words, we constructed gene function profiles of the microorganisms in the samples and “mapped” the composition of the sequenced colonies into the database to analyze the metabolic functions of the colonies.

### 2.7 RNA-seq analysis

RNA was extracted with TRIzol reagent and assayed on a 1% agarose gel. Total RNA samples were assessed for purity, concentration and integrity before further analysis. High-throughput sequencing of multiple samples was performed using the Illumina Hiseq sequencing platform.The raw reads were filtered by removing adapter and poly-N sequences and inferior quality reads from the raw reads. HISAT2 software was used to quickly and accurately compare the clean reads with the reference genome to obtain the localization information of the reads on the reference genome. The levels of quantitative gene expression were estimated by determining the fragments per kilobase of transcript per million fragments mapped. Limma was used to identify differentially expressed genes (DEGs) in different groups. *P* < 0.05 and |log2FC| > 0.5 were defined as significant differences. Gene ontology (GO) function, Kyoto encyclopedia of genes and genomes (KEGG) pathway Enrichment Analysis and Gene Set Enrichment Analysis (GSEA) were performed using DEGs.

### 2.8 Single-cell RNA sequencing data resources and analysis methods

The UC-associated single-cell transcriptome dataset GSE182270 was downloaded from the GEO database (https://www.ncbi.nlm.nih.gov/geo/) (24). Data quality control was based on gene counts > 200 and < 2500 for each cell, and a percentage of mitochondrial genes < 5%. Subsequently, the “vst” method was used in the FindVariableFeatures function to select the top 2000 highly variable expression genes. The principal component analysis (PCA) and clustering analysis of data were achieved through the uniform manifold approximation and projection (UMAP) of RunPCA, FindClusters, and RunUMAP functions. In addition, we applied Single R V1.4.1 and CellMarker 2.0 to annotate cell types (25). Finally, single-cell functional enrichment analysis and intercellular communication analysis were performed using R package “irGSEA” and “Cellchat”, respectively (26). Functional scoring of cimifugin related regulatory genes obtained from transcriptome was performed using “AUCell” and “irGSEA” packages to obtain cimifugin related target cells and visualize them.

### 2.9 Cell culture and treatment

Mouse monocyte-macrophage RAW 264.7 cells were procured from Pricella (Wuhan, China). These cells were cultured in Dulbecco’s modified Eagle’s medium (DMEM; Gibco, Grand Island, USA) supplemented with 10% Fetal bovine serum (FBS; Gibco) and 1% penicillin-streptomycin (Solarbio, Beijing, China). Culturing was conducted at 5% CO2 and 37°C in a humidified incubator. The experimental design proceeded as follows: the LPS group was treated with 1 μg/mL LPS (Solarbio) for 24 h. The control group was treated with PBS (Gibco). The cimifugin intervention group was divided into three groups according to the concentration gradient (50mg/L, 100mg/L, 200mg/L), and each group was treated with the above concentrations of LPS and cimifugin for 24 hours. For the pathway inhibition assay, cells were treated with 8 μM Upadacitinib (a selective JAK1 inhibitor, Cayman) for 1 hour before administration.

### 2.10 Enzyme-linked immunosorbent assay

Colon and lung tissues were prepared as homogenates and the concentration of total protein was determined by BCA protein assay kit. Culture supernatants of cells corresponding to each group in the *in vitro* study were also collected. The concentrations of IL-6 (88–7064), IL-1β (88–7013), and TNF-α (88–7324) in the above samples were determined using a commercial ELISA kit according to the manufacturer’s instructions (Thermo Fisher Scientific Inc.).

### 2.11 Statistical analyses

Statistical analysis was conducted using GraphPad Prism 8.0 (GraphPad, San Diego, CA, USA). Data are presented as the mean ± Standard error of mean (SEM). Significant differences between two groups were evaluated using a two-tailed unpaired Student’s t-test, whereas the differences among multiple groups were analyzed using one-way analysis of variance (ANOVA) followed by Dunnett’s *post hoc* multiple comparison’s test. All results were considered statistically significant at *P* < 0.05.

## 3 Results

### 3.1 Cimifugin ameliorates colitis symptoms and lung injury

**Figure 1:**
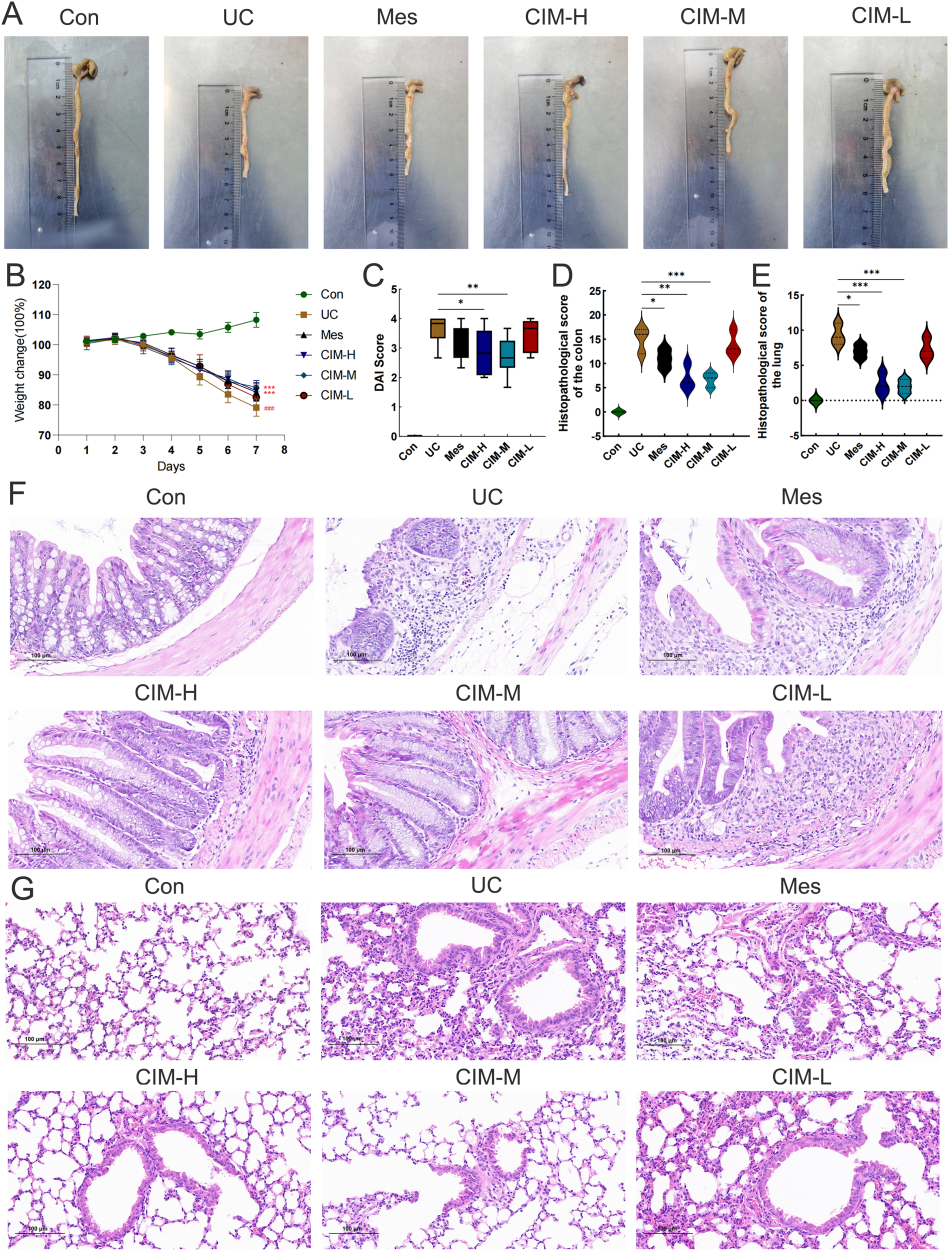
Cimifugin ameliorates DSS-induced colitis and lung injury. **(A)** Differences in colon length between different groups (n = 6). **(B)** Changes in body weight of mice in different groups. **(C)** DAI scores of mice in different groups. **(D, E)** Differences in histopathologic scores of colon and lung between different groups. **(F)** Representative images of HE staining of the colon in different groups of mice (n = 3). **(G)** Representative images of HE staining of lungs in different groups of mice (n = 3). Con, Control group; UC, Ulcerative colitis group; Mes, Mesalazine group; CIM-H, High-dose cimifugin group; CIM-M, Medium-dose cimifugin group; CIM-L, Low-dose cimifugin group. ^###^ *P* < 0.001, compared with Con group. *P < 0.5; **P < 0.01; ***P < 0.001, compared with DSS group.

**Figure 2:**
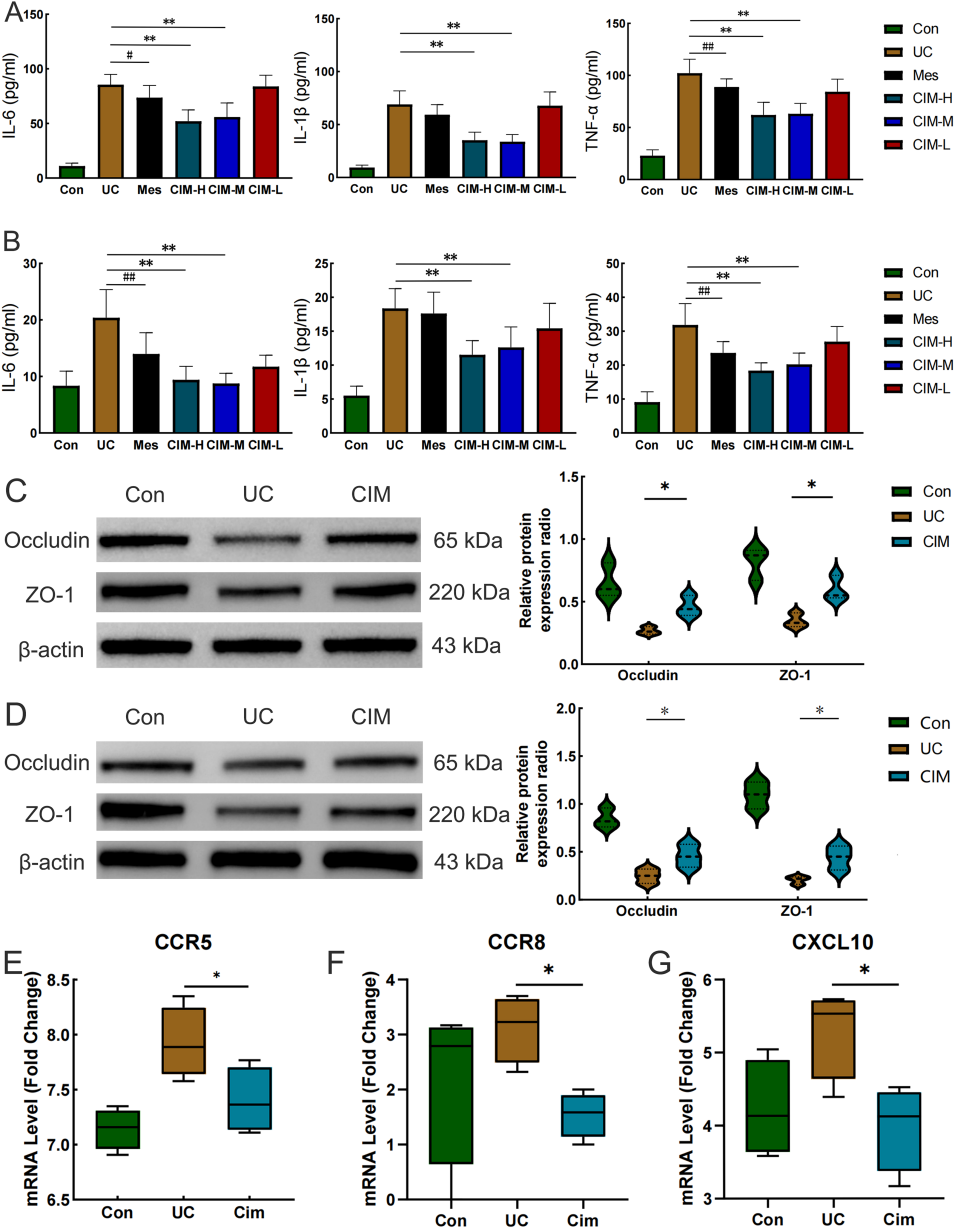
Cimifugin ameliorates DSS-induced colonic epithelial barrier damage and lung inflammation. **(A)** Effect of Cimifugin on IL-6, IL-1β, and TNF-α levels in colonic tissue (n = 6). **(B)** Effect of Cimifugin on IL-6, IL-1β, and TNF-α levels in lung tissues (n = 6). **(C)** Expression levels of Occludin and ZO-1 proteins in the colon of different groups of mice (n = 3). **(D)** Expression levels of Occludin and ZO-1 proteins in the lung of different groups of mice (n = 3). **(E–G)** The mRNA expression levels of CCR5, CCR8 and CXCL10 in lung tissues of different groups of mice (n = 4). Con, Control group; UC, Ulcerative colitis group; Mes, Mesalazine group; CIM-H, High-dose cimifugin group; CIM-M, Medium-dose cimifugin group; CIM-L, Low-dose cimifugin group; IL-6, Interleukin - 6; IL-1β, Interleukin-1 beta; TNF-α, Tumor necrosis factor-alpha; ZO-1, Zonula Occludens-1; CCR5, C-C chemokine receptor type 5; CCR8, C-C chemokine receptor type 8; CXCL10, C-X-C motif chemokine ligand 10. ^#^ *P* < 0.05, ^##^ *P* < 0.01, compared with Mes group. *P < 0.5; **P < 0.01, compared with DSS group.

In this study, the most widely used colonic length, body weight change and DAI score were selected to confirm the ameliorative effect of cimifugin in UC mice. Colon length was significantly shorter in the UC group of mice compared to normal mice, while this effect was significantly suppressed in the CIM-H and CIM-M groups (**Figure 1A**). The body weight of DSS-treated mice was significantly decreased, and this trend was significantly attenuated by high and medium doses of cimifugin compared to the UC group, with no difference between the two, but the effect of low doses of cimifugin was not significant (**Figure 1B**). In addition, DAI scores were elevated in the UC group compared with the normal group, indicating successful induction of the UC model. Both high and medium doses of cimifugin reduced the DAI scores of mice, which was more significant in the CIM-M group, but mesalazine and low-dose cimifugin had no significant effect on this (**Figure 1C**). Histopathology showed that the intestines of mice in the UC group exhibited extensive epithelial damage, inflammatory cell infiltration, abnormal crypts and localized ulceration. More importantly, the present study similarly observed that UC mice developed significant lung inflammation and tissue damage, including widened alveolar septa, interstitial edema and alveolar collapse, and the presence of inflammatory cell infiltration centered on the airways. As expected, high and medium doses of cimifugin inhibited DSS-induced disruption of the intestinal mucosa, and both intestinal inflammation and epithelial barrier were ameliorated to varying degrees, airway inflammation and alveolar septal widening were also significantly ameliorated, and histopathological scores of both lungs and colon were significantly reduced (**Figures 1D–G**). In addition, quantitative analysis of several key inflammatory factors (IL-6, IL-1β, and TNF-α) also showed that both high and medium doses of cimifugin significantly down-regulated their concentrations in the lungs and colon, with non-significant differences between groups (**Figures 2A, B**). It is worth mentioning that both high and medium doses of cimifugin did not cause significant pathological damage to the liver and kidney of mice, indicating a high safety profile (**Supplementary Figures S1A, B**). In summary, we focused primarily on the pharmacologic effects of medium-dose cimifugin, taking into account health economic costs and potential drug side effects. Occludin and ZO-1 are two tight junction-associated proteins that play important roles in maintaining the integrity of intestinal and lung epithelia. The expression levels of these proteins were both reduced in the UC group compared with the control group, and cimifugin significantly reversed the DSS-induced decrease in protein expression (**Figures 2C, D**). In lung tissues, cimifugin mainly down-regulated the mRNA expression levels of classical inflammatory factors such as CCR5, CCR8 and CXCL10 (**Figures 2E–G**). In conclusion, the above results suggest that cimifugin significantly improved the severity of ulcerative colitis and its associated lung injury.

### 3.2 Cimifugin ameliorates gut flora dysbiosis in DSS-induced UC models

**Figure 3:**
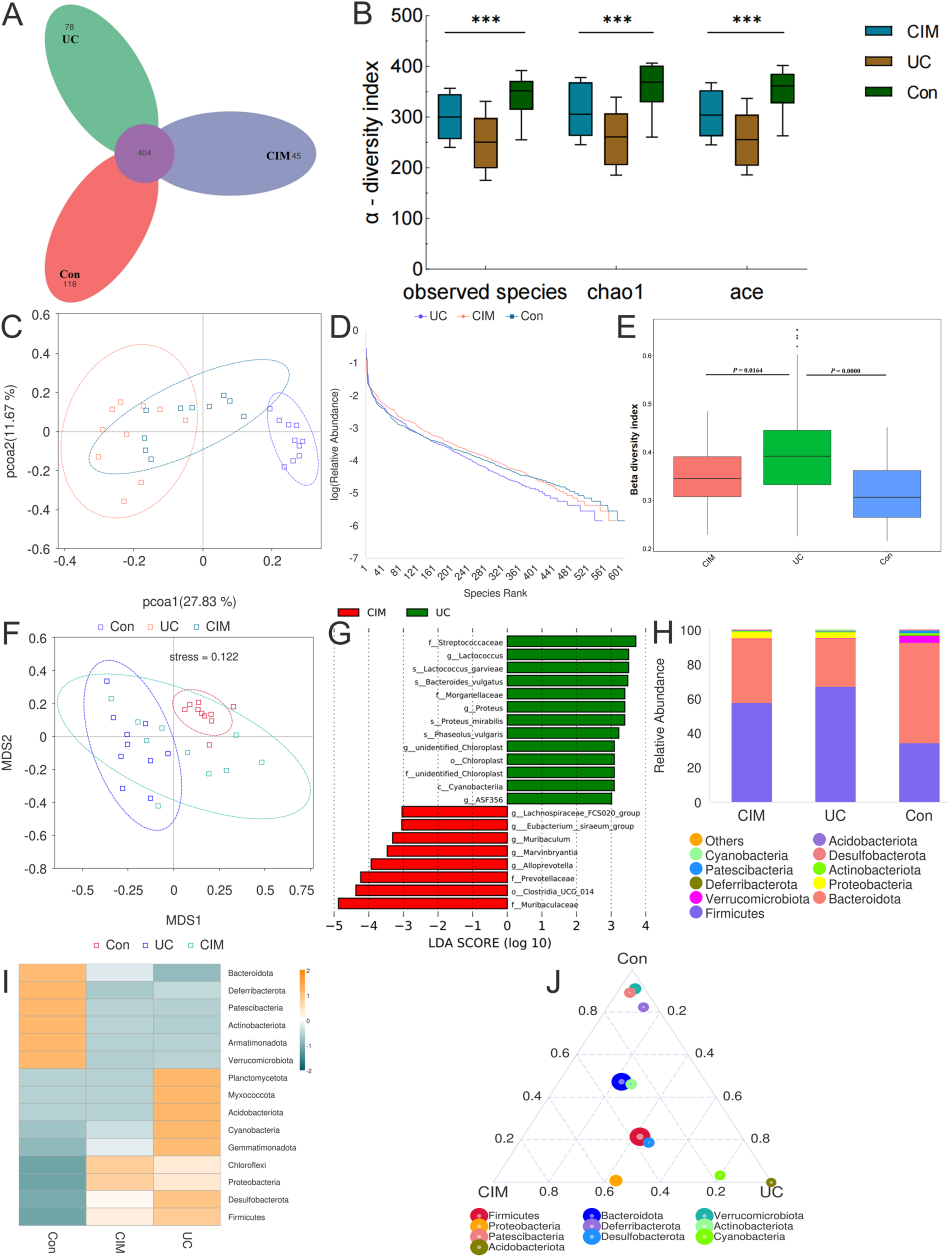
Cimifugin remodeled the gut microbiota composition in mice with DSS-induced ulcerative colitis. **(A)** Petal plot demonstrating the difference in the number of OTUs between the 3 groups. **(B)** α-diversity analysis based on multiple methods such as observed-species, Chao1, and ACE. **(C)** Bray-Curtis principal coordinate analysis (PCoA) analysis of gut microbiota based on the OTU level among three groups. **(D)** Three groups’ Rank Abundance curves. In the horizontal direction, species richness is reflected by the width of the curve, the higher the species richness, the larger the span of the curve on the horizontal axis; in the vertical direction, the smoothness of the curve reflects the homogeneity of the species in the samples, the flatter the curve, the more homogeneous the species distribution. **(E)** Between-group comparison of β-diversity indices. **(F)** NMDS based on the differences in gut microbiota among the three groups. **(G)** Histogram of the distribution of LDA values in the CIM and UC groups. **(H)** Classification and composition ratio of the gut microbiota of mice in the three groups at the phylum level. **(I)** Based on the species annotation and abundance information of the intestinal microbiota of mice in the three groups at the phylum level, the heat map was obtained by clustering using the maximum ranking method. **(J)** Select the top 10 species with average abundance in the three groups of samples at the phylum classification level, and generate a ternaryplot reflecting the difference of dominant species among the three groups of samples. Con, Control group; UC, Ulcerative colitis group; CIM, Cimifugin group. N = 10, ***P < 0.001.

**Figure 4:**
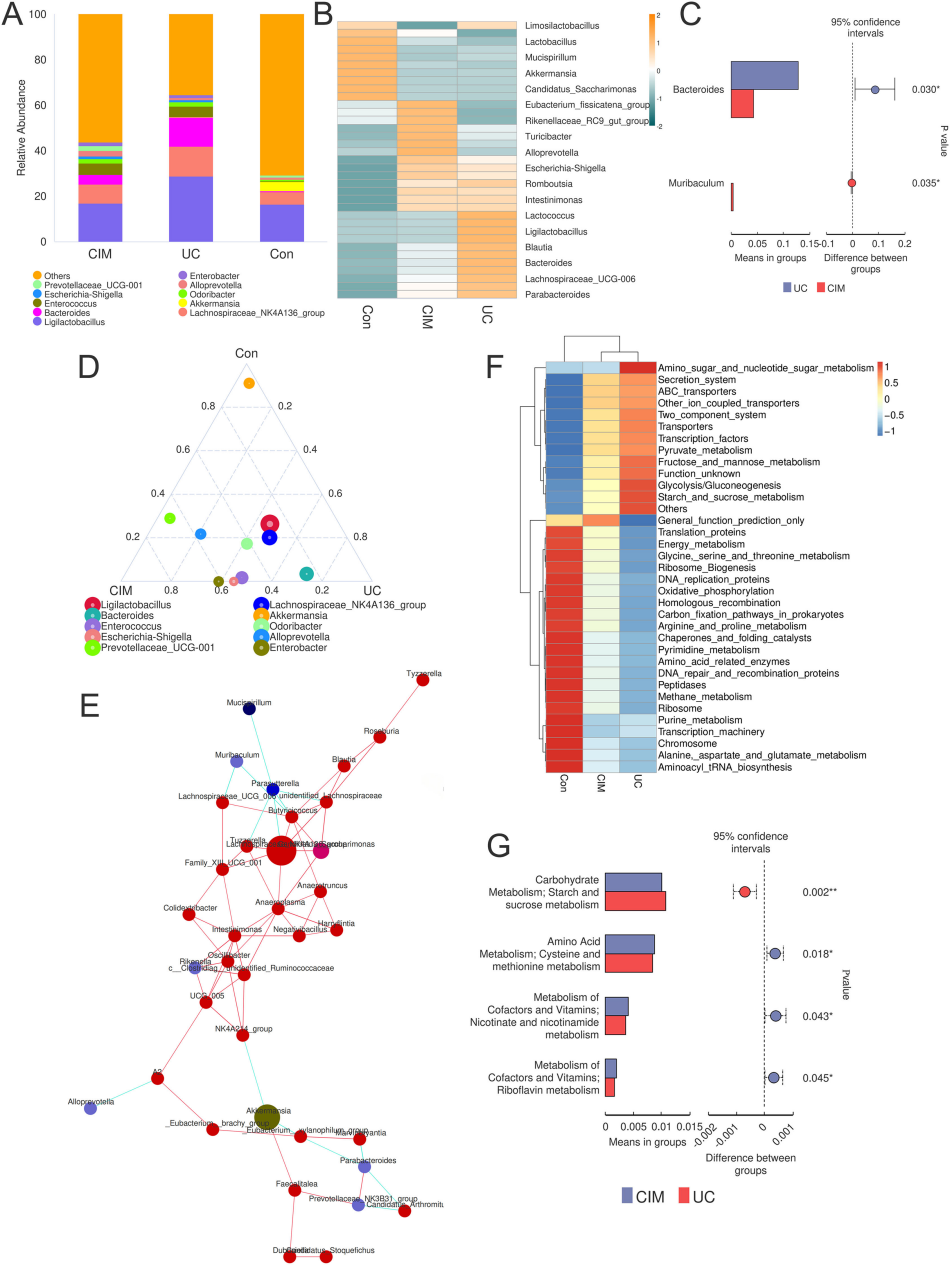
Cimifugin alters the genomic function of the gut microbiota. **(A)** Taxonomic and compositional ratios of the gut microbiota of mice in the three groups at the genus level. **(B)** Heatmap obtained by clustering the gut microbiota of mice in the three groups based on species annotation and abundance information at the genus level using the maximum value ranking method. **(C)** Species with significant differences at the genus level between the UC and CIM groups. **(D)** Species that ranked in the top 10 in average abundance at the genus classification level for the three groups of samples were selected to generate a ternaryplot reflecting the differences in dominant species among the three groups of samples. **(E)** Species correlation network plot showing dominant species that dominate under the action of cimifugin and the clusters of species that are closely related to them. **(F)** Heatmap of the relative abundance of microbial genome functions among different groups obtained based on PICRUSt analysis. **(G)** Metabolic processes in the presence of significant differences between the UC and CIM groups. Con, Control group; UC, Ulcerative colitis group; CIM, Cimifugin group. N = 10, *P < 0.5; **P < 0.01.

Considering the significant contribution of intestinal flora dysbiosis to the progression of UC, we assessed the effect of cimifugin on the diversity and composition of intestinal flora by 16S rRNA amplicon sequencing. A total of 643 OTUs were obtained in the normal control, UC and cimifugin-treated groups, of which 404 were common to all 3 groups. There were 116, 78, and 45 unique OTUs in the normal control, UC, and cimifugin-treated groups, respectively (**Figure 3A**). cimifugin administration attenuated the DSS-induced decrease in bacterial diversity and abundance as shown by the α-diversity index represented by observed-species, Chao1, and ACE (**Figure 3B**). Indeed, PCoA analysis revealed that the cimifugin-treated group had a more similar microbial community structure to the normal control group (**Figure 3C**), which was confirmed by the Rank Abundance curve, i.e., the CIM group had a longer and smoother span of the curve relative to the UC group, suggesting higher species richness and a more homogeneous species distribution (**Figure 3D**). Comparison of beta diversity indices between groups confirmed that cimifugin significantly improved the beta diversity of the samples, and NMDS also found that the CIM group was closer to the normal group (**Figures 3E, F**). In terms of species composition, LEfSe analysis showed that the UC and CIM groups had different taxa and suggested that *Muribaculum*, *Marvinbryantia*, *Alloprevotella*, *Prevotellaceae*, and *Muribaculaceae* might mediate the interfering effects of cimifugin on UC (**Figure 3G**). Specifically, at the phylum level, cimifugin inhibited the increase in the number of *Planctomycetota*, *Myxococcota*, *Acidobacteriota*, *Cyanobacteria*, *Gemmatimonadota*, *Desulfobacterota*, and *Firmicutes* and increased abundance of *Bacteroidota* (**Figures 3H, I**). **Figure 3J** demonstrates the differences in dominant species at the phylum level among the three groups, with *Acidobacteriota* identified as the dominant species in the UC group and *Proteobacteria* as the dominant species in the CIM group. At the genus level, cimifugin suppressed the abundance of *Lactococcus*, *Ligilactobacillus*, *Blautia*, *Bacteroides*, *Parabacteroides*, and *Romboutsia* and increased the abundance of *Lactobacillus* (**Figures 4A, B**). T-test revealed that the abundance of Bacteroides was significantly lower and Muribaculum was significantly higher in the CIM group compared to the UC group (*P* < 0.05, **Figure 4C**). **Figure 4D** demonstrates the differences in dominant species at the genus level among the three groups, where *Bacteroides* was identified as the dominant species in the UC group, while *Prevotellaceae*, *Alloprevotella*, and *Enterobacter* were the dominant species in the CIM group. After cimifugin treatment, *Parasutterella*, *Butyricoccus*, *Anaeroplasma*, and *Akkermansia* were identified as dominant species in the CIM group that dominated the interactions and played unique as well as important roles in maintaining the stability of microbial community structure and functioning in this environment (**Figure 4E**). Meanwhile, cimifugin also significantly affected the genomic function of the colony. cimifugin down-regulated the activity of ion coupled transporters, gene transcription processes, and glycolysis, and up-regulated the enrichment of processes such as energy metabolism, amino acid metabolism, and homologous recombination (**Figure 4F**). Interestingly, alterations in metabolism-related pathways were an important feature of the CIM group. cimifugin significantly enhanced cysteine, methionine, riboflavin, niacin, and nicotinamide metabolism in the colony and down-regulated carbohydrate metabolism processes (*P* < 0.05, **Figure 4G**). Taken together, cimifugin may attenuate DSS-induced intestinal dysbiosis by altering intestinal microbiota composition and metabolic functions.

### 3.3 Cimifugin targets JAK1/STAT1 signaling pathway to improve ulcerative colitis-related lung injury

**Figure 5:**
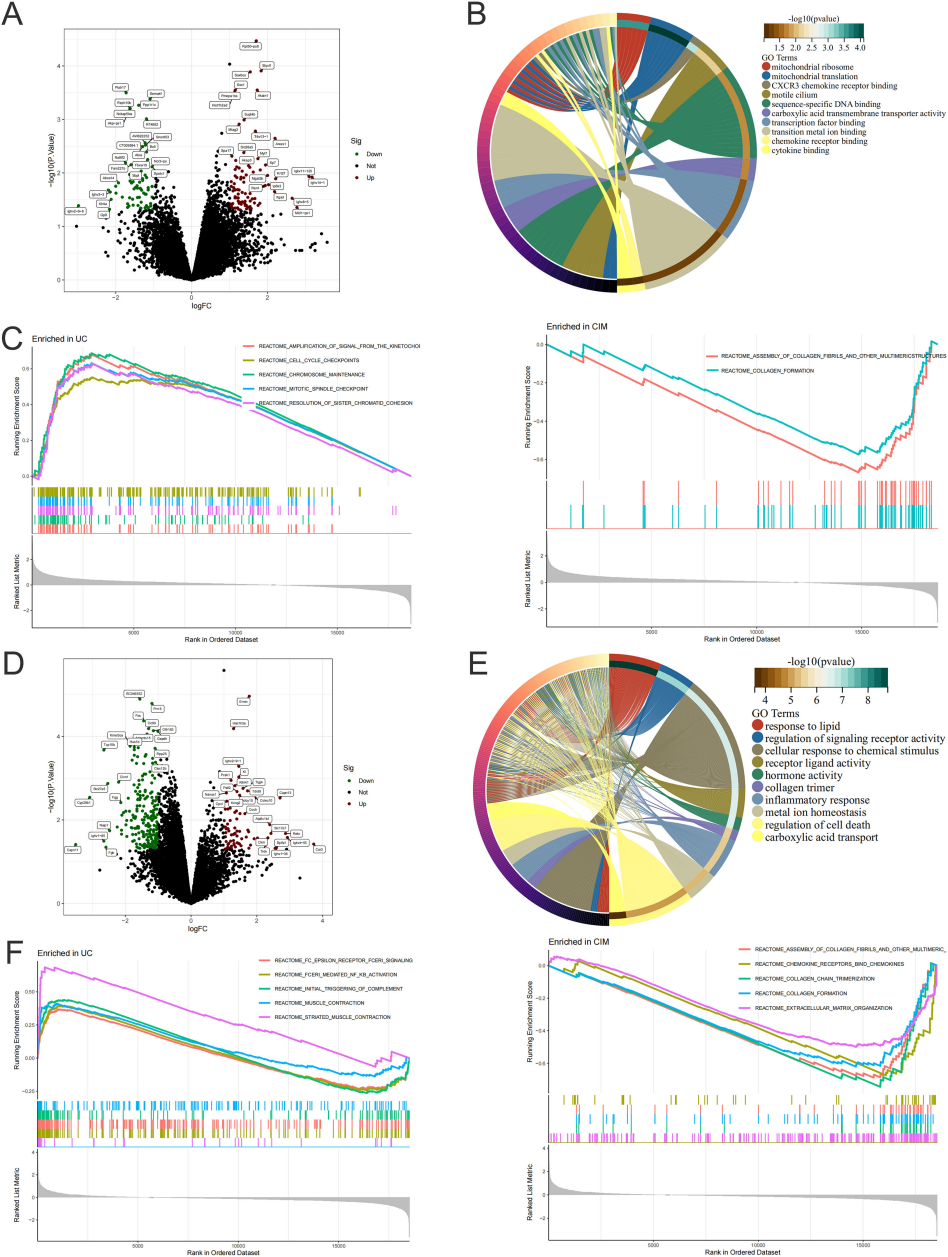
Cimifugin alters gene expression in colon and lung tissue. **(A)** Volcano plot demonstrating up- and down-regulated genes in the colon of the CIM group compared to the UC group. **(B)** GO enrichment analysis of DEGs in the colon. **(C)** GSEA analysis of DEGs in the colon of both groups. **(D)** Volcano plots demonstrating up- and down-regulated genes in lung tissues of the CIM group compared with the UC group. **(E)** GO enrichment analysis of DEGs in lung. **(F)** GSEA analysis of DEGs in lung tissues of both groups. N = 4.

**Figure 6:**
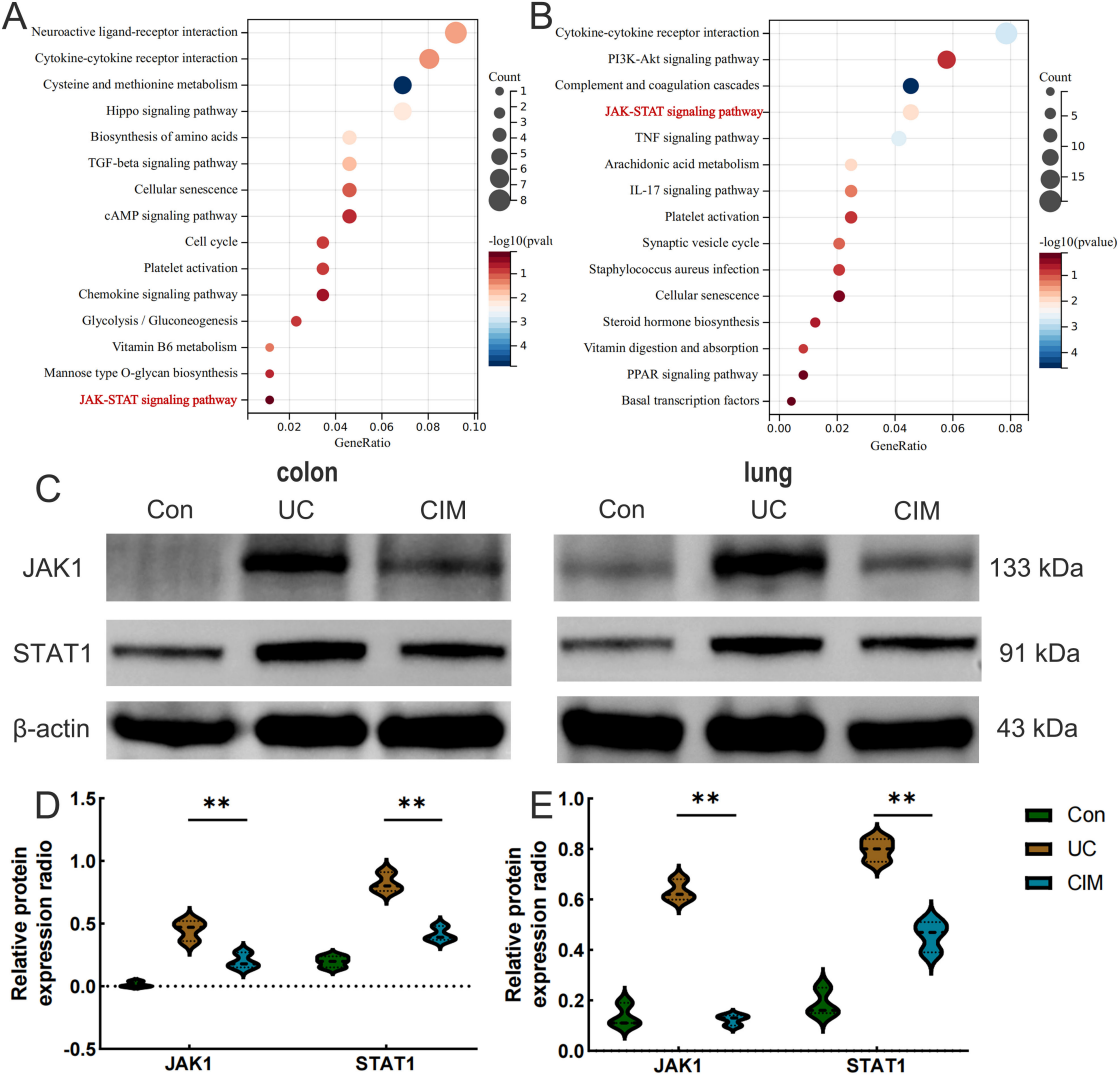
Cimifugin affects JAK1/STAT1 protein expression in colon and lung tissues of UC mouse models. **(A)** KEGG enrichment analysis of DEGs in colon. **(B)** KEGG enrichment analysis of DEGs in lung tissues. **(C–E)** Expression levels of JAK1 and STAT1 proteins in colon and lung tissues of different groups of mice (n = 3). Con, Control group; UC, Ulcerative colitis group; CIM,: Cimifugin group; JAK1, Janus kinase 1; STAT1, Signal Transducer and Activator of Transcription 1. **P < 0.01.

To further investigate the signaling mechanism of cimifugin to improve ulcerative colitis-related lung injury, we performed RNA-seq analysis on colon and lung tissues separately. Transcriptome analysis showed that 228 DEGs existed between colon tissues of CIM and UC groups (**Figure 5A**), and GO analysis revealed that DEGs were mainly concentrated in mitochondrial translation, CXCR3 chemokine receptor binding, motile cilium, carboxylic acid transmembrane transporter activity, transition metal ion binding, chemokine receptor binding, cytokine binding, and other processes closely related to UC (**Figure 5B**). Compared with normal mice, cell cycle checkpoints, mitotic spindle checkpoints, and other pathways related to cell division were upregulated in the UC group, while collagen formation was downregulated in the CIM group (**Figure 5C**). In lung tissues, 605 DEGs existed between the CIM and UC groups (**Figure 5D**), and GO analysis revealed that DEGs were mainly focused on response to lipid, cellular response to chemical stimulus, hormone activity, collagen trimer and regulation of cell death, while regulation of signaling receptor activity, inflammatory response, metal ion homeostasis and carboxylic acid transport are biological processes shared with the colon, suggesting that the above processes may be common target processes for cimifugin to ameliorate ulcerative colitis-related lung injury (**Figure 5E**). GSEA similarly validated these results and found that FCϵRI and NF-κB-mediated signaling processes were up-regulated in lung tissues of UC mice compared to normal mice, whereas cimifugin mainly down-regulated the activity of collagen synthesis and chemokine signaling pathways (**Figure 5F**). In the colon, cimifugin mainly acts on cytokine-cytokine receptor interaction, cysteine and methionine metabolism, Hippo signaling pathway, TGF-β signaling pathway, cellular senescence, glycolysis, and JAK-STAT signaling pathway (**Figure 6A**), whereas in the lung, cimifugin mainly affected cytokine-cytokine receptor interaction, PI3K-Akt signaling pathway, TNF signaling pathway, JAK-STAT signaling pathway, IL-17 signaling pathway, *Staphylococcus aureus* infection, cellular senescence, PPAR signaling pathway (**Figure 6B**). Considering that the JAK-STAT signaling pathway was mentioned in both tissues and that tofacitinib, which is a pan-Janus kinase inhibitor, was also shown to be effective in UC, we explored the effect of cimifugin on the JAK1/STAT1 signaling pathway (27). The expression of both JAK1 and STAT1 was up-regulated in lung and colon tissues in the DSS group compared with the control group, whereas the expression of both JAK1 and STAT1 proteins was significantly suppressed by the administration of cimifugin (**Figures 6C–E**).

### 3.4 Cimifugin inhibits macrophage polarization to M1 in the colon and lungs

**Figure 7:**
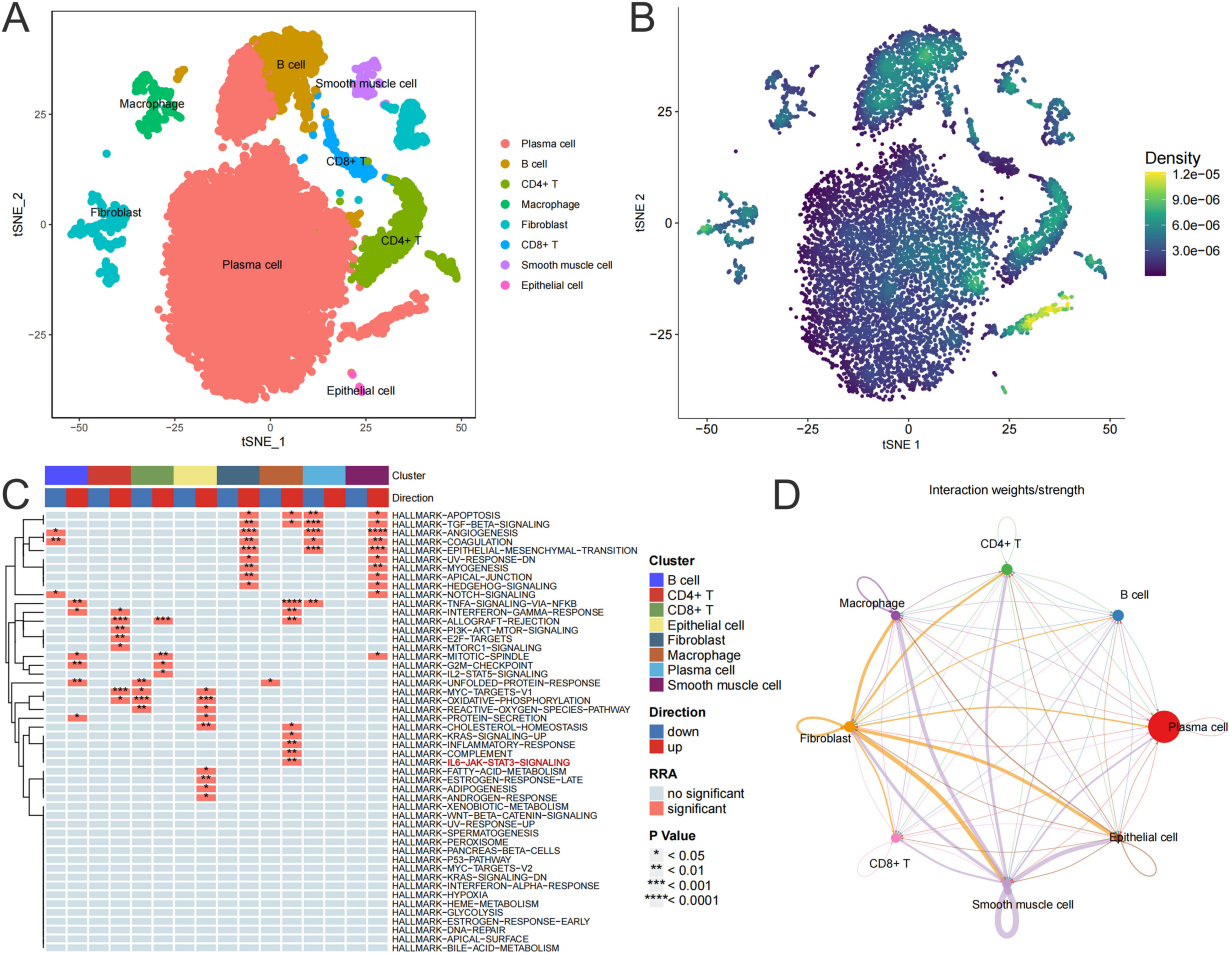
M1 macrophage may be a target cell for cimifugin intervention in UC. **(A)** TSNE used for dimensionality reduction, cell classification, and definition. **(B)** Scoring the regulatory genes related to cimifugin and mapping them to transcriptional changes in various types of cells to obtain the target cells most likely to be intervened by cimifugin. **(C)** Changes in the enrichment pathways of marker gene sets for each cell subpopulation in UC. **(D)** Inference of intercellular communication network strength in UC sample colon tissue, with the thickness of the lines representing the strength.

**Figure 8:**
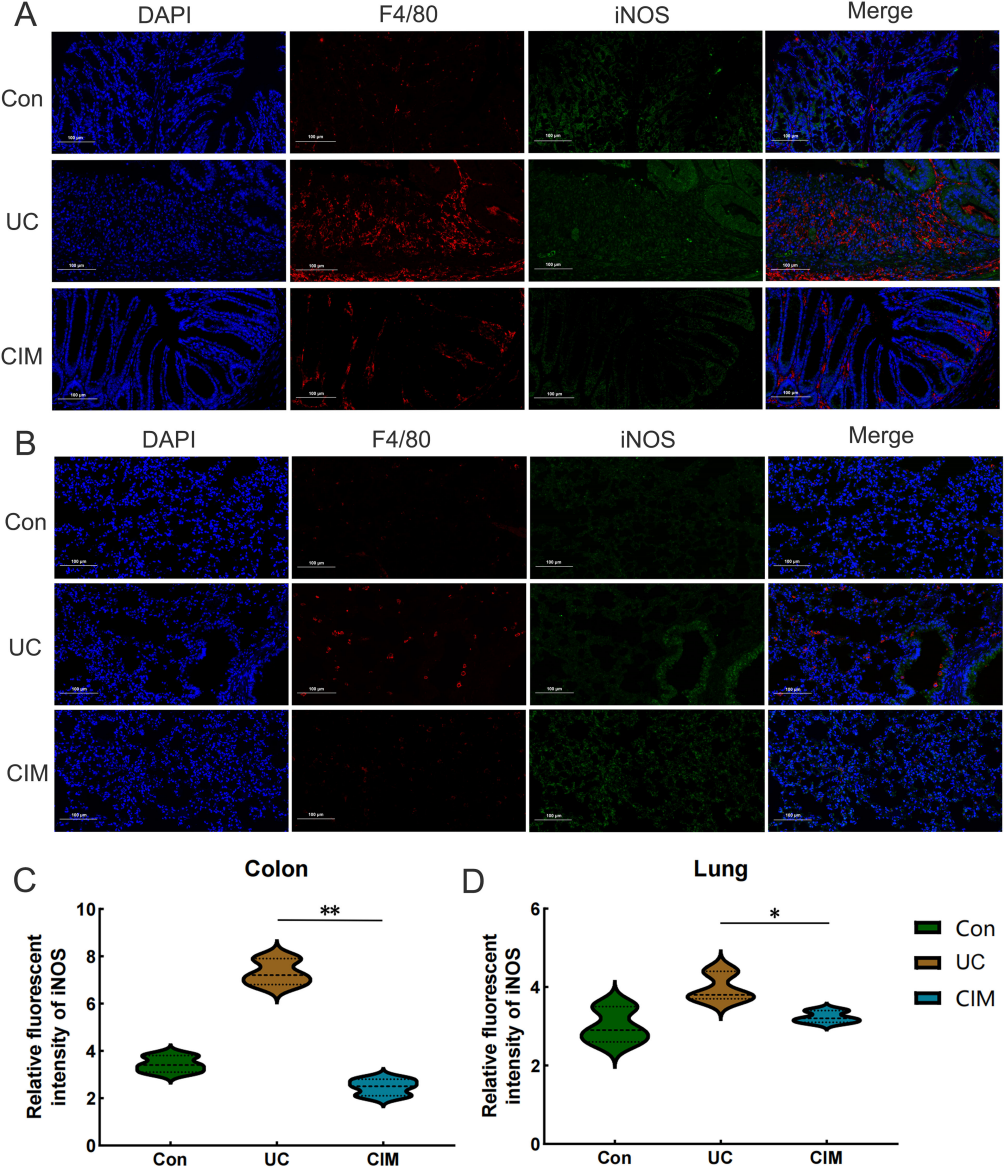
*In vivo* experiments confirmed the centrality of M1 macrophages in Cimifugin intervention in UC and its associated lung injury. **(A)** Immunofluorescence revealed the degree of M1 macrophage infiltration in the colon of different groups of mice. **(B)** Immunofluorescence revealed the degree of M1 macrophage infiltration in the lungs of different groups of mice. **(C, D)** Quantitative analysis results of immunofluorescence in colon and lung tissues (n = 3). Con, Control group; UC, Ulcerative colitis group; CIM, Cimifugin group; iNOS, inducible nitric oxide synthase; DAPI, 4’,6-diamidino-2-phenylindole. *P < 0.5; **P < 0.01.

UC is typically an immune-disordered disease, so we combined scRNA-seq data from the colon of UC patients with RNA-seq data from the colon tissue of the CIM group to look for immune cells that might be targeted by cimifugin. Gene expression markers were used to classify all cells into eight major subpopulations, including plasma cells, B cells, CD4^+^ T cells, macrophage, fibroblast, CD8^+^ T cells, smooth muscle cells, and epithelial cells (**Figure 7A**). The cimifugin-related regulatory genes obtained from transcriptome analysis were functionally scored and found to mainly fit the gene transcriptional changes in plasma cell, B cell, CD4^+^ T cell and macrophage (**Figure 7B**). Among them, macrophage had the most significant UC-related features, such as inflammatory response and cell death, which were mainly reflected in the activation of TNF-α/NF-κB signaling pathway, γ-interferon signaling pathway, and apoptosis, etc., and there was a higher intensity of intercellular communication between macrophage and other cell types (**Figures 7C, D**). Most importantly, among the eight major subpopulations, JAK/STAT was activated only in macrophage, suggesting that M1 macrophage, which exhibits a pro-inflammatory phenotype, may be a target cell for cimifugin. Immunofluorescence showed that compared with normal mice, the infiltration of M1 macrophages in the colon tissue of the UC group was significantly increased, while cimifugin downregulated the abundance of M1 macrophages. More importantly, this synchronous phenomenon was also found in lung tissue (**Figures 8A, B**). These findings are consistent with previous analysis of scRNA seq, suggesting that macrophages may be the target cells mediating the protective effect of cimifugin on UC and its associated lung injury (**Figures 8C, D**).

### 3.5 Cimifugin regulates macrophage polarization to resist inflammation by inhibiting JAK1/STAT1 pathway activation

**Figure 9:**
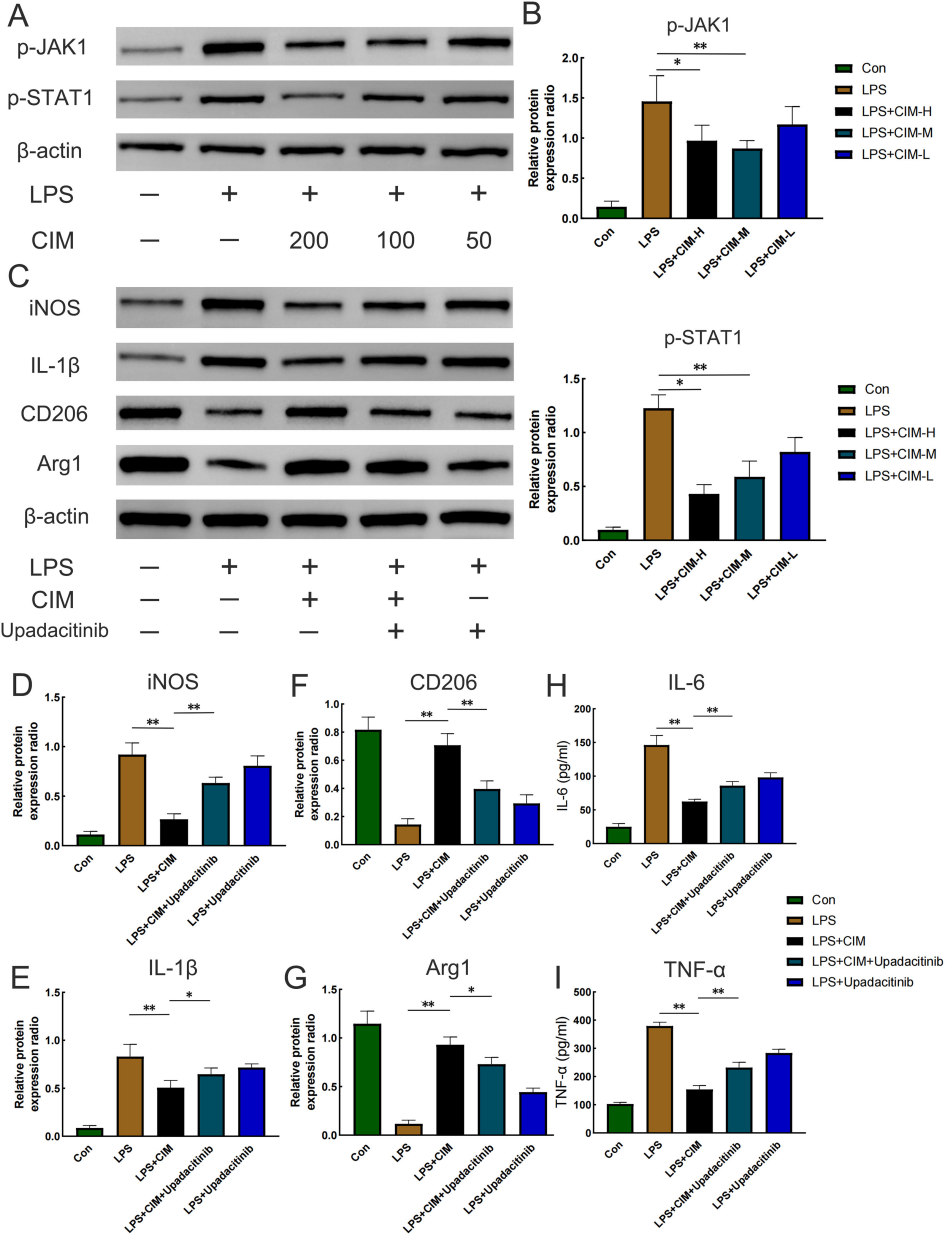
Cimifugin regulates M1 polarization in macrophages by inhibiting activation of the JAK1/STAT1 pathway. **(A)** Effects of different concentrations of cimifugin on the phosphorylation of JAK1 and STAT1 in an *in vitro* model. **(B)** Analysis of grayscale values corresponding to p-JAK1 and p-STAT1. **(C)** Expression levels of the corresponding markers after treatment with cimifugin and Upadacitinib. **(D–G)** Analysis of grayscale values corresponding to various markers (iNOS, IL-1β, Arg1, CD206). **(H, I)** Concentrations of IL-6 and TNF-α in different groups. Con, Control group; LPS, Lipopolysaccharide; Cim, Cimifugin; CIM-H, High-dose cimifugin group; CIM-M, Medium-dose cimifugin group; CIM-L, Low-dose cimifugin group; IL-6, Interleukin - 6; IL-1β, Interleukin-1 beta; TNF-α, Tumor necrosis factor-alpha; JAK1, Janus kinase 1; STAT1, Signal Transducer and Activator of Transcription 1; iNOS, inducible nitric oxide synthase; CD206, Cluster of Differentiation 206; Arg1, arginase 1. N = 3, *P < 0.5; **P < 0.01.

Next, we investigated how cimifugin regulates macrophage polarization. Considering that lung and colon are co-enriched for the JAK1/STAT1 pathway at the transcriptional level, we investigated the effect of cimifugin on the activation of the JAK1/STAT1 pathway during macrophage polarization. As expected, LPS promoted the phosphorylation of JAK1 and STAT1 in macrophages, whereas cimifugin inhibited this process (**Figures 9A, B**). Considering that there was no significant difference between the effects of treatment at 100 mg/L and treatment at 200 mg/L on the expression of p-JAK1 and p-STAT1 in macrophages, we chose to observe the correlation between activation of the JAK1/STAT1 pathway and cimifugin-regulated macrophage polarization under the intervention of the 100 mg/L concentration. *In vitro*, LPS promoted M1 polarization in macrophages. However, cimifugin treatment down-regulated the expression of M1 polarization-related markers (IL-1β, iNOS) and up-regulated M2 polarization-related markers (CD206, Arg1), respectively. The modulation of macrophage polarization-related proteins by cimifugin was counteracted after specific inhibition of JAK1 activation using Upadacitinib (**Figures 9C–G**). Specifically, the LPS + CIM + Upadacitinib group exhibited higher levels of iNOS and IL-1β expression, as well as lower levels of Arg1, CD206 expression, than the LPS + CIM group. In addition, the concentrations of IL-6 and TNF-α detected by ELISA showed expression trends consistent with those of M1 polarization-related markers after Upadacitinib treatment (**Figures 9H, I**).

## 4 Discussion

UC is an IBD characterized by intestinal inflammation, abdominal pain, and blood in the stool.Even in the absence of respiratory symptoms, patients with UC may have abnormalities on pulmonary function tests, radiology, and histopathology (28, 29). These untreated airway inflammations can cause irreversible destruction of the airways and therefore require stable, effective long-term pharmacologic interventions (30). In this study, we demonstrated the therapeutic efficacy of cimifugin in UC-related lung injury. By combining RNA-seq and 16S rRNA sequencing of both organs, these findings enabled us to construct a comprehensive network from a more holistic perspective, which in turn enhanced our understanding of the deeper mechanisms of cimifugin ameliorating UC-related lung injury and further demonstrated that its mechanism is related to the inhibition of the JAK1/STAT1 pathway and M1 macrophage-mediated inflammatory states in the colon and lungs, as well as ameliorating gut microbiota disruption.

The respiratory tract and gastrointestinal tract are both rich in epithelial cells, goblet cells, submucosal glands, and lymphoid tissue, and the same embryological origin has led researchers to consider the possibility that both may suffer the same type of damage (31). Whether it’s an antigen, cytokine storm, or a specific pathogen, all may play a potential role in UC-related lung injury. The breakdown of the intestinal epithelial barrier is a prominent feature of UC. The injury of the intestinal mucosa and intestinal inflammation make the tight connection between the epithelium fail, and the microbial antigens in the intestinal cavity leak, thus promoting the abnormal immune response of the whole body (32). For example, memory T cells are exposed to specific antigens in the inflamed intestinal mucosa and are induced to express large amounts of C-C chemokine receptor 3 and C-X-C chemokine receptor 5 (33). Subsequently, they can migrate to broncho-associated lymphoid tissue, where they express more chemokine receptors. Leakage of microbial antigens into the peripheral circulation also induces activation of tissue-resident macrophages, which induce neutrophil-mediated inflammation by secreting a range of cytokines (34). Indeed, current studies on UC-related lung injury have also focused on exploring the disruption of immune homeostasis by M1 macrophage (3). In our study, CXCL10 was found for the first time to be a differentially expressed chemokine between lung tissues of the UC and normal groups, whereas CXCL10^+^ macrophage has been shown to be enriched in the colon of patients with UC, exhibiting pro-inflammatory gene expression characterized by interferon-related responses (35). CCR5 has also been shown to enhance macrophage migration and infiltration in the lung (36). Although there are no studies on the effects of cimifugin on lung diseases, demonstrated that cimifugin reduced the expression of the above chemokines and their receptors, inhibited the enrichment of M1 macrophage in the colon and lungs, and upregulated the expression of tight junction proteins, such as ZO-1 and Occludin, suggesting that cimifugin restores the structure and function of the epithelial barrier and maintains immune homeostasis in both organs.

Although pan-JAK-inhibitor tofacitinib, also known as IBD, was effective in UC patients but not in Crohn’s patients, suggesting that the JAK/STAT signaling pathway may induce more severe inflammation in UC. From published data, JAK inhibitors provide significant clinical benefit in both acute exacerbation and maintenance phases of UC (37). JAK inhibitors show better therapeutic efficacy compared to traditional drugs such as TNF-α monoclonal antibodies and mesalazine (38). JAK inhibitors provide benefit even if the patient has already been treated with a TNF-α monoclonal antibody (39). Adverse events associated with JAK inhibitors are generally controllable and dose-dependent, so the lowest effective dose should be used during the maintenance phase of treatment (40). Although cimifugin has not yet been used in clinical practice, in light of this principle, an appropriate dose range should be explored for cimifugin, which is also a JAK inhibitor. The expression and activation levels of STAT1 in colon tissues of UC patients were significantly increased, which was mainly attributed to infiltrating peripheral neutrophils and monocytes/macrophages (41). In fact, JAK/STAT signaling plays a decisive role in the differentiation of monocytes into macrophages, and macrophage activation is thought to be a driver of intestinal inflammation (42). Studies have shown that inhibition of the JAK1/STAT1 signaling pathway causes macrophages to shift from a pro-inflammatory M1-like phenotype to an M2-like phenotype that inhibits inflammation, ultimately leading to an accelerated recovery of colon inflammation. Based on previously published studies, this study selected three doses within the dose range of 12.5-50 mg/kg as the research subjects and found that both concentrations of 25 mg/kg and 50 mg/kg of cimifugin could significantly inhibit the activation of the JAK1/STAT1 pathway. It indicates that it is a promising inhibitor of the JAK/STAT target (17, 43, 44). Considering that the four different molecules of the JAK family and the seven members of the STAT family have great plasticity and complexity in UC and its associated lung injury, future research should focus on identifying the contribution of each member to further develop highly selective targeted drugs (45, 46).

Many studies have shown that gut flora plays a key role in the pathogenesis of UC, and that improvements in gut flora composition and metabolic patterns can lead to disease remission or even cure (47, 48). In the present study, we demonstrated that cimifugin significantly reversed the intestinal flora disruption in a mouse model of UC, suggesting that the protective effect of cimifugin may be related to its modulation of the intestinal microbiota. Notably, cimifugin treatment significantly increased the abundance of *Lactobacillus* and *Butyricoccus* was identified as the dominant species in the CIM group. Previous studies have shown reduced colonization of *Lactobacillus* as a potential anti-inflammatory bacterium in a mouse model of UC. *Lactobacillus* can alleviate colitis by specifically increasing the proportion of intestinal macrophages and IL-10 secretion through a mechanism that promotes the conversion of macrophages to M2 macrophages and release of IL-10 (49). At the same time, this effect was associated with the promotion of protective acetate production by *Lactobacillus johnsonii (* 50). *Butyricicoccus* is a butyrate-producing clostridial cluster IV genus whose numbers are reduced in the stool of UC patients (51). *Butyricoccus* can reverse the inflammation-induced elevation of CLDN1 protein levels by secreting butyrate, which in turn maintains the integrity of the epithelial barrier. In addition, these *Butyricoccus*-derived butyric acids were able to promote the expression of IL-10, ARG1, and CD206 in macrophages in UC and induced their polarization to M2 (52). These connections between microbiota and their metabolites, which have been intensively studied, undoubtedly provide a powerful note for understanding the triad of cimifugin, the JAK1/STAT1 pathway, and macrophage differentiation. We also observed a significant reduction in the abundance of pathogenic *Bacteroides* after cimifugin treatment. A cohort study including 73 patients with UC found that patients with active UC had high levels of *Bacteroides*-derived proteases in their intestines, and these proteases directly induced barrier dysfunction (53). In addition, cimifugin significantly enhances the metabolism of cysteine, methionine, riboflavin, niacin and nicotinamide in the intestinal microbiota. Among them, supplementation of methionine has been proven to alleviate the severity of the disease and inhibit the expression of inflammation-related genes (54). The mechanism may be related to the abnormal activation of M1 macrophages and the alteration of the production pattern of short-chain fatty acids (55). Riboflavin, on the other hand, attenuates colitis by inhibiting neutrophil infiltration, modulating oxidative stress, and ameliorating DNA damage, as evidenced by a reduction in the ulcer index and the inflammation index (56, 57). niacin has been suggested to protect mice from DSS-induced colitis in a D prostanoid receptor 1-dependent manner, partly through the mechanism of inhibition of pro-inflammatory gene expression in macrophages (58). Together, these results suggest that cimifugin may maintain the stability of the internal environment by increasing the abundance of potentially beneficial bacteria and decreasing pathogenic bacteria.

The study has several flaws. First, due to the lack of lung single-cell transcriptome sequencing samples from UC-related lung injury, this study could not confirm the key target cells of cimifugin in human lung tissue. Second, although cimifugin has been identified as a key species in improving gut microbiota dysregulation, its regulation of microbial metabolic patterns remains unclear. Moreover, UC can often turn into a chronic course, and the composition of the gut microbiota, metabolic patterns, and immune disordered states during the stable phase may be different from those during the acute phase, which needs to be identified in future studies. Finally, other possible mechanisms of action of cimifugin in the intervention of UC-related lung injury suggested by transcriptomics still need further experimental verification.

## 5 Conclusions

Taken together, our work suggests that oral administration of cimifugin ameliorates DSS-induced colitis and its associated lung inflammation, which is associated with its inhibition of chemokine release, reduction of M1 macrophage infiltration and down-regulation of JAK1/STAT1 protein expression. On the other hand, cimifugin may ameliorate the imbalance of the gut microbiota by regulating the colonization of specific microorganisms. Our findings may contribute to a better understanding of the molecular events and immune microenvironment of UC-related lung injury and highlight the therapeutic potential of cimifugin in related diseases.

## Data Availability

The datasets presented in this study can be found in online repositories. The names of the repository/repositories and accession number(s) can be found in the article/**Supplementary Material**.
