# Supplementary material for: Cimifugin ameliorates ulcerative colitis-related lung injury by modulating the JAK1/STAT1 signaling pathway and macrophage M1 polarization
Source: Front Immunol. 2025 Jul 1;16:1551892. doi: 10.3389/fimmu.2025.1551892 (PMC12259561; doi:10.3389/fimmu.2025.1551892)
Supplement: Supplementary file 2 [file Table1.docx]

https://www.jianguoyun.com/p/DU45MXkQqYCoChiDoOgFIAA
