# Supplementary material for: Cimifugin ameliorates ulcerative colitis-related lung injury by modulating the JAK1/STAT1 signaling pathway and macrophage M1 polarization
Source: Front Immunol. 2025 Jul 1;16:1551892. doi: 10.3389/fimmu.2025.1551892 (PMC12259561; doi:10.3389/fimmu.2025.1551892)
Supplement: Supplementary file 3 [file Table2.docx]

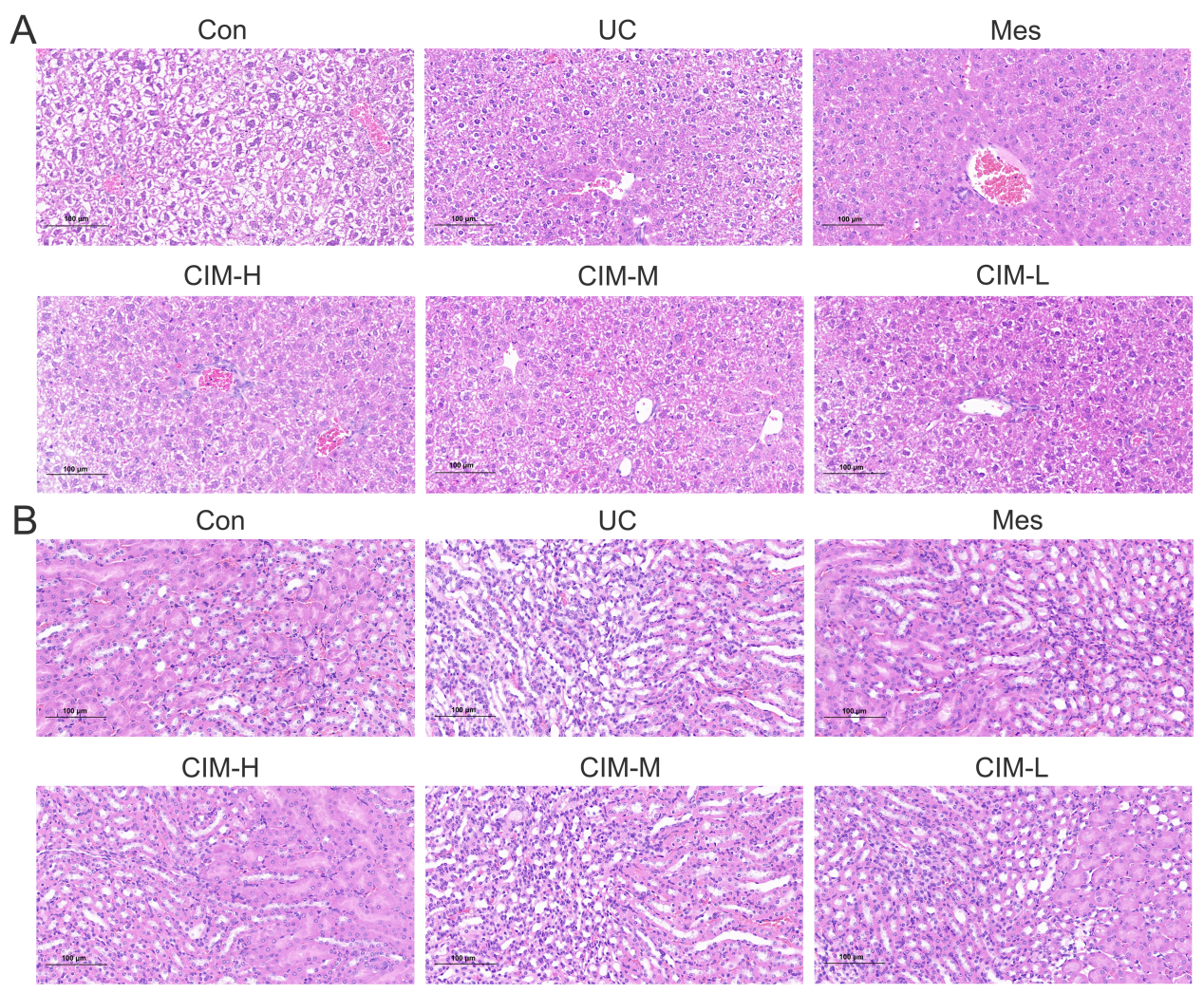


Figure S1. Hepatorenal toxicity profile of Cimifugin. (A) Representative images of HE staining of the livers of different groups of mice. (B) Representative images of HE staining of kidneys from different groups of mice.
